# Supplementary material for: Presyncodon, a Web Server for Gene Design with the Evolutionary Information of the Expression Hosts
Source: Int J Mol Sci. 2018 Dec 4;19(12):3872. doi: 10.3390/ijms19123872 (PMC6321224; doi:10.3390/ijms19123872)
Supplement: Supplementary file 1 [file ijms-19-03872-s001.pdf]

Table S1. The Genome IDs used in this study

Species Name	Genome NO	IDs	Species Name	Genome NO	IDs
**E.coli**	1	GCA_000005845.2	E.coli	219	GCA_001890245.1
**E.coli**	2	GCA_000007445.1	E.coli	220	GCA_001890265.1
**E.coli**	3	GCA_000008865.1	E.coli	221	GCA_001890285.1
**E.coli**	4	GCA_000009565.2	E.coli	222	GCA_001890305.1
**E.coli**	5	GCA_000010245.1	E.coli	223	GCA_001890325.1
**E.coli**	6	GCA_000010385.1	E.coli	224	GCA_001890345.1
**E.coli**	7	GCA_000010485.1	E.coli	225	GCA_001890365.1
**E.coli**	8	GCA_000010745.1	E.coli	226	GCA_001900295.1
**E.coli**	9	GCA_000010765.1	E.coli	227	GCA_001900315.1
**E.coli**	10	GCA_000013265.1	E.coli	228	GCA_001900335.1
**E.coli**	11	GCA_000013305.1	E.coli	229	GCA_001900355.1
**E.coli**	12	GCA_000014845.1	E.coli	230	GCA_001900375.1
**E.coli**	13	GCA_000017745.1	E.coli	231	GCA_001900395.1
**E.coli**	14	GCA_000017765.1	E.coli	232	GCA_001900415.1
**E.coli**	15	GCA_000017985.1	E.coli	233	GCA_001900435.1
**E.coli**	16	GCA_000019385.1	E.coli	234	GCA_001900455.1
**E.coli**	17	GCA_000019425.1	E.coli	235	GCA_001900475.1
**E.coli**	18	GCA_000019645.1	E.coli	236	GCA_001900495.1
**E.coli**	19	GCA_000021125.1	E.coli	237	GCA_001900515.1
**E.coli**	20	GCA_000022225.1	E.coli	238	GCA_001900535.1
**E.coli**	21	GCA_000022345.1	E.coli	239	GCA_001900555.1
**E.coli**	22	GCA_000022665.2	E.coli	240	GCA_001900575.1
**E.coli**	23	GCA_000023365.1	E.coli	241	GCA_001900595.1
**E.coli**	24	GCA_000023665.1	E.coli	242	GCA_001900615.1
**E.coli**	25	GCA_000025165.1	E.coli	243	GCA_001900635.1
**E.coli**	26	GCA_000025745.1	E.coli	244	GCA_001900655.1
**E.coli**	27	GCA_000026245.1	E.coli	245	GCA_001900675.1
**E.coli**	28	GCA_000026265.1	E.coli	246	GCA_001900695.1
**E.coli**	29	GCA_000026285.1	E.coli	247	GCA_001900715.1
**E.coli**	30	GCA_000026345.1	E.coli	248	GCA_001900735.1
**E.coli**	31	GCA_000026545.1	E.coli	249	GCA_001900775.1
**E.coli**	32	GCA_000027125.1	E.coli	250	GCA_001900795.1
**E.coli**	33	GCA_000091005.1	E.coli	251	GCA_001900815.1
**E.coli**	34	GCA_000147855.3	E.coli	252	GCA_001900835.1
**E.coli**	35	GCA_000148365.1	E.coli	253	GCA_001900885.1
**E.coli**	36	GCA_000148605.1	E.coli	254	GCA_001900905.1
**E.coli**	37	GCA_000183345.1	E.coli	255	GCA_001900925.1
**E.coli**	38	GCA_000184185.1	E.coli	256	GCA_001900945.1

**E.coli**	39	GCA_000210475.1	E.coli	257	GCA_001900965.1
**E.coli**	40	GCA_000212715.2	E.coli	258	GCA_001900985.1
**E.coli**	41	GCA_000219515.3	E.coli	259	GCA_001901005.1
**E.coli**	42	GCA_000227625.1	E.coli	260	GCA_001901025.1
**E.coli**	43	GCA_000233875.1	E.coli	261	GCA_001901045.1
**E.coli**	44	GCA_000233895.1	E.coli	262	GCA_001901065.1
**E.coli**	45	GCA_000245515.1	E.coli	263	GCA_001901085.1
**E.coli**	46	GCA_000257275.1	E.coli	264	GCA_001901105.1
**E.coli**	47	GCA_000258025.1	E.coli	265	GCA_001901125.1
**E.coli**	48	GCA_000258145.1	E.coli	266	GCA_001901145.1
**E.coli**	49	GCA_000262125.1	E.coli	267	GCA_001901165.1
**E.coli**	50	GCA_000270105.1	E.coli	268	GCA_001901185.1
**E.coli**	51	GCA_000284495.1	E.coli	269	GCA_001901215.1
**E.coli**	52	GCA_000285655.3	E.coli	270	GCA_001901315.1
**E.coli**	53	GCA_000299255.1	E.coli	271	GCA_001901365.1
**E.coli**	54	GCA_000299455.1	E.coli	272	GCA_001901405.1
**E.coli**	55	GCA_000299475.1	E.coli	273	GCA_001901425.1
**E.coli**	56	GCA_000332755.1	E.coli	274	GCA_001901445.1
**E.coli**	57	GCA_000350185.1	E.coli	275	GCA_001901465.1
**E.coli**	58	GCA_000468515.1	E.coli	276	GCA_001932515.1
**E.coli**	59	GCA_000474035.1	E.coli	277	GCA_001936315.1
**E.coli**	60	GCA_000493755.1	E.coli	278	GCA_001969285.1
**E.coli**	61	GCA_000499485.1	E.coli	279	GCA_001999185.1
**E.coli**	62	GCA_000520035.1	E.coli	280	GCA_002007705.1
**E.coli**	63	GCA_000520055.1	E.coli	281	GCA_002009315.1
**E.coli**	64	GCA_000597845.1	E.coli	282	GCA_002011945.1
**E.coli**	65	GCA_000599625.1	E.coli	283	GCA_002011965.1
**E.coli**	66	GCA_000599645.1	E.coli	284	GCA_002011985.1
**E.coli**	67	GCA_000599665.1	E.coli	285	GCA_002012005.1
**E.coli**	68	GCA_000599685.1	E.coli	286	GCA_002012025.1
**E.coli**	69	GCA_000599705.1	E.coli	287	GCA_002012045.1
**E.coli**	70	GCA_000662395.1	E.coli	288	GCA_002012065.1
**E.coli**	71	GCA_000671295.1	E.coli	289	GCA_002012085.1
**E.coli**	72	GCA_000714595.1	E.coli	290	GCA_002012105.1
**E.coli**	73	GCA_000725265.1	E.coli	291	GCA_002012125.1
**E.coli**	74	GCA_000725305.1	E.coli	292	GCA_002012145.1
**E.coli**	75	GCA_000730345.1	E.coli	293	GCA_002012165.1
**E.coli**	76	GCA_000732965.1	E.coli	294	GCA_002012185.1
**E.coli**	77	GCA_000743255.1	E.coli	295	GCA_002012205.1
**E.coli**	78	GCA_000750555.1	E.coli	296	GCA_002012225.1
**E.coli**	79	GCA_000784925.1	E.coli	297	GCA_002012245.1

**E.coli**	80	GCA_000800215.1	E.coli	298	GCA_002012265.1
**E.coli**	81	GCA_000800765.1	E.coli	299	GCA_002012305.1
**E.coli**	82	GCA_000800845.2	E.coli	300	GCA_002024865.1
**E.coli**	83	GCA_000801165.1	E.coli	301	GCA_002055605.1
**E.coli**	84	GCA_000801185.2	E.coli	302	GCA_002055635.1
**E.coli**	85	GCA_000801205.1	E.coli	303	GCA_002056065.1
**E.coli**	86	GCA_000803705.1	E.coli	304	GCA_002056145.1
**E.coli**	87	GCA_000813165.1	E.coli	305	GCA_002056635.1
**E.coli**	88	GCA_000814145.2	E.coli	306	GCA_002057245.1
**E.coli**	89	GCA_000819645.1	E.coli	307	GCA_002057355.1
**E.coli**	90	GCA_000829985.1	E.coli	308	GCA_002058765.1
**E.coli**	91	GCA_000830035.1	E.coli	309	GCA_002078275.1
**E.coli**	92	GCA_000831565.1	E.coli	310	GCA_002078295.1
**E.coli**	93	GCA_000833145.1	E.coli	311	GCA_002079225.1
**E.coli**	94	GCA_000833635.2	E.coli	312	GCA_002090355.1
**E.coli**	95	GCA_000931565.1	E.coli	313	GCA_002105735.1
**E.coli**	96	GCA_000952955.1	E.coli	314	GCA_002116715.1
**E.coli**	97	GCA_000953515.1	E.coli	315	GCA_002118095.1
**E.coli**	98	GCA_000967155.1	E.coli	316	GCA_002142675.1
**E.coli**	99	GCA_000968515.1	E.coli	317	GCA_002142695.1
**E.coli**	100	GCA_000971615.1	E.coli	318	GCA_002142715.1
**E.coli**	101	GCA_000974405.1	E.coli	319	GCA_002156825.1
**E.coli**	102	GCA_000974465.1	E.coli	320	GCA_002156845.1
**E.coli**	103	GCA_000974505.1	E.coli	321	GCA_002157245.1
**E.coli**	104	GCA_000974535.1	E.coli	322	GCA_002163655.1
**E.coli**	105	GCA_000974575.1	E.coli	323	GCA_002163695.1
**E.coli**	106	GCA_000974825.1	E.coli	324	GCA_002163935.1
**E.coli**	107	GCA_000974865.1	E.coli	325	GCA_002163955.1
**E.coli**	108	GCA_000974885.1	E.coli	326	GCA_002180055.1
**E.coli**	109	GCA_000981485.1	E.coli	327	GCA_002180095.1
**E.coli**	110	GCA_000986765.1	E.coli	328	GCA_002180135.1
**E.coli**	111	GCA_000987875.1	E.coli	329	GCA_002180195.1
**E.coli**	112	GCA_000988355.1	E.coli	330	GCA_002180215.1
**E.coli**	113	GCA_000988385.1	E.coli	331	GCA_002180275.1
**E.coli**	114	GCA_000988425.1	E.coli	332	GCA_002192275.1
**E.coli**	115	GCA_000988445.1	E.coli	333	GCA_002192295.1
**E.coli**	116	GCA_000988465.1	E.coli	334	GCA_002193095.1
**E.coli**	117	GCA_001007915.1	E.coli	335	GCA_002196475.1
**E.coli**	118	GCA_001020945.2	E.coli	336	GCA_002196495.1
**E.coli**	119	GCA_001021005.2	E.coli	337	GCA_002201835.1
**E.coli**	120	GCA_001021595.1	E.coli	338	GCA_002202175.1

**E.coli**	121	GCA_001021615.1	E.coli	339	GCA_002208865.1
**E.coli**	122	GCA_001021635.1	E.coli	340	GCA_002209105.1
**E.coli**	123	GCA_001029125.1	E.coli	341	GCA_002211725.1
**E.coli**	124	GCA_001039415.1	E.coli	342	GCA_002214205.1
**E.coli**	125	GCA_001043215.1	E.coli	343	GCA_002214745.1
**E.coli**	126	GCA_001051135.1	E.coli	344	GCA_002215095.1
**E.coli**	127	GCA_001183645.1	E.coli	345	GCA_002215115.1
**E.coli**	128	GCA_001183665.1	E.coli	346	GCA_002215155.1
**E.coli**	129	GCA_001183685.1	E.coli	347	GCA_002220215.1
**E.coli**	130	GCA_001276585.2	E.coli	348	GCA_002220265.1
**E.coli**	131	GCA_001280325.1	E.coli	349	GCA_002237285.1
**E.coli**	132	GCA_001280345.1	E.coli	350	GCA_002237305.1
**E.coli**	133	GCA_001280385.1	E.coli	351	GCA_002237325.1
**E.coli**	134	GCA_001280405.1	E.coli	352	GCA_900092615.1
**E.coli**	135	GCA_001307215.1	E.coli	353	GCA_900174625.1
**E.coli**	136	GCA_001308065.1	B.subtilis	1	GCA_000009045.1
**E.coli**	137	GCA_001308125.1	B.subtilis	2	GCA_000146565.1
**E.coli**	138	GCA_001308165.1	B.subtilis	3	GCA_000186745.1
**E.coli**	139	GCA_001420935.1	B.subtilis	4	GCA_000209795.2
**E.coli**	140	GCA_001420955.1	B.subtilis	5	GCA_000227465.1
**E.coli**	141	GCA_001442495.1	B.subtilis	6	GCA_000227485.1
**E.coli**	142	GCA_001455385.1	B.subtilis	7	GCA_000293765.1
**E.coli**	143	GCA_001469815.1	B.subtilis	8	GCA_000321395.1
**E.coli**	144	GCA_001485455.1	B.subtilis	9	GCA_000328745.1
**E.coli**	145	GCA_001513615.1	B.subtilis	10	GCA_000338735.1
**E.coli**	146	GCA_001513635.1	B.subtilis	11	GCA_000344745.1
**E.coli**	147	GCA_001513655.1	B.subtilis	12	GCA_000349795.1
**E.coli**	148	GCA_001515725.1	B.subtilis	13	GCA_000497485.1
**E.coli**	149	GCA_001542675.2	B.subtilis	14	GCA_000523045.1
**E.coli**	150	GCA_001544635.1	B.subtilis	15	GCA_000699465.1
**E.coli**	151	GCA_001558995.2	B.subtilis	16	GCA_000699525.1
**E.coli**	152	GCA_001559615.2	B.subtilis	17	GCA_000706705.1
**E.coli**	153	GCA_001559635.1	B.subtilis	18	GCA_000737405.1
**E.coli**	154	GCA_001559655.1	B.subtilis	19	GCA_000772125.1
**E.coli**	155	GCA_001559675.1	B.subtilis	20	GCA_000772165.1
**E.coli**	156	GCA_001566335.1	B.subtilis	21	GCA_000772205.1
**E.coli**	157	GCA_001566615.1	B.subtilis	22	GCA_000782835.1
**E.coli**	158	GCA_001566635.1	B.subtilis	23	GCA_000789275.1
**E.coli**	159	GCA_001566655.1	B.subtilis	24	GCA_000789295.1
**E.coli**	160	GCA_001566675.1	B.subtilis	25	GCA_000816805.1
**E.coli**	161	GCA_001577325.1	B.subtilis	26	GCA_000827065.1

**E.coli**	162	GCA_001593565.1	B.subtilis	27	GCA_000953615.1
**E.coli**	163	GCA_001610755.1	B.subtilis	28	GCA_000959025.1
**E.coli**	164	GCA_001612475.1	B.subtilis	29	GCA_000971925.1
**E.coli**	165	GCA_001612495.1	B.subtilis	30	GCA_000973605.1
**E.coli**	166	GCA_001617565.1	B.subtilis	31	GCA_001015095.1
**E.coli**	167	GCA_001618325.1	B.subtilis	32	GCA_001037985.1
**E.coli**	168	GCA_001618345.2	B.subtilis	33	GCA_001465815.1
**E.coli**	169	GCA_001618365.1	B.subtilis	34	GCA_001534785.1
**E.coli**	170	GCA_001644725.1	B.subtilis	35	GCA_001541905.1
**E.coli**	171	GCA_001644745.1	B.subtilis	36	GCA_001565875.1
**E.coli**	172	GCA_001651925.1	B.subtilis	37	GCA_001596535.1
**E.coli**	173	GCA_001651945.1	B.subtilis	38	GCA_001597265.1
**E.coli**	174	GCA_001651965.2	B.subtilis	39	GCA_001604995.1
**E.coli**	175	GCA_001660565.1	B.subtilis	40	GCA_001660525.1
**E.coli**	176	GCA_001660585.1	B.subtilis	41	GCA_001697265.1
**E.coli**	177	GCA_001663075.1	B.subtilis	42	GCA_001703495.1
**E.coli**	178	GCA_001663475.1	B.subtilis	43	GCA_001704095.1
**E.coli**	179	GCA_001675145.1	B.subtilis	44	GCA_001720505.1
**E.coli**	180	GCA_001677475.1	B.subtilis	45	GCA_001746575.1
**E.coli**	181	GCA_001677495.1	B.subtilis	46	GCA_001747445.1
**E.coli**	182	GCA_001677515.1	B.subtilis	47	GCA_001808235.1
**E.coli**	183	GCA_001678925.1	B.subtilis	48	GCA_001889385.1
**E.coli**	184	GCA_001678965.1	B.subtilis	49	GCA_001889625.1
**E.coli**	185	GCA_001679985.1	B.subtilis	50	GCA_001890405.1
**E.coli**	186	GCA_001682305.2	B.subtilis	51	GCA_001902555.1
**E.coli**	187	GCA_001683435.1	B.subtilis	52	GCA_002055965.1
**E.coli**	188	GCA_001693315.1	B.subtilis	53	GCA_002072735.1
**E.coli**	189	GCA_001693635.1	B.subtilis	54	GCA_002096095.1
**E.coli**	190	GCA_001695515.1	B.subtilis	55	GCA_002163815.1
**E.coli**	191	GCA_001721125.1	B.subtilis	56	GCA_002173695.1
**E.coli**	192	GCA_001721205.1	B.subtilis	57	GCA_002173715.1
**E.coli**	193	GCA_001721225.1	B.subtilis	58	GCA_002201955.1
**E.coli**	194	GCA_001721525.1	B.subtilis	59	GCA_002201995.1
**E.coli**	195	GCA_001723505.1	B.subtilis	60	GCA_002202035.1
**E.coli**	196	GCA_001735705.1	B.subtilis	61	GCA_002202055.1
**E.coli**	197	GCA_001750845.1	B.subtilis	62	GCA_002216085.1
**E.coli**	198	GCA_001753445.1	S.cerevisiae	1	GCA_000146045.2
**E.coli**	199	GCA_001753465.1	S.cerevisiae	2	GCA_000149365.1
**E.coli**	200	GCA_001753485.1	S.cerevisiae	3	GCA_000181435.1
**E.coli**	201	GCA_001753505.1	S.cerevisiae	4	GCA_000182175.1
**E.coli**	202	GCA_001753525.1	S.cerevisiae	5	GCA_000182315.2

**E.coli**	203	GCA_001753545.1	S.cerevisiae	6	GCA_000190195.1
**E.coli**	204	GCA_001753565.1	S.cerevisiae	7	GCA_000190215.1
**E.coli**	205	GCA_001806265.1	S.cerevisiae	8	GCA_000190235.1
**E.coli**	206	GCA_001806285.1	S.cerevisiae	9	GCA_000190255.1
**E.coli**	207	GCA_001860505.1	S.cerevisiae	10	GCA_000218975.1
**E.coli**	208	GCA_001865295.1	S.cerevisiae	11	GCA_000260735.1
**E.coli**	209	GCA_001874485.1	S.cerevisiae	12	GCA_000269885.1
**E.coli**	210	GCA_001886535.1	S.cerevisiae	13	GCA_000325965.1
**E.coli**	211	GCA_001886555.1	S.cerevisiae	14	GCA_000326005.1
**E.coli**	212	GCA_001886575.1	S.cerevisiae	15	GCA_000568005.1
**E.coli**	213	GCA_001886755.1	S.cerevisiae	16	GCA_000568055.1
**E.coli**	214	GCA_001886895.1	S.cerevisiae	17	GCA_000568295.1
**E.coli**	215	GCA_001886935.1	S.cerevisiae	18	GCA_000568365.1
**E.coli**	216	GCA_001888075.1	S.cerevisiae	19	GCA_001634645.1
**E.coli**	217	GCA_001890205.1	S.cerevisiae	20	GCA_001983315.1
**E.coli**	218	GCA_001890225.1			

Table S2. The codon selection at the N- or C-terminal

Amino acid pair in the N_terminal	E.coli	B.subtilis	S.cerevisiae
MA	ATGGCA	ATGGCA	ATGGCT
MC	ATGTGT	ATGTGT	ATGTGT
MD	ATGGAT	ATGGAT	ATGGAT
ME	ATGGAA	ATGGAA	ATGGAA
MF	ATGTTT	ATGTTT	ATGTTT
MG	ATGGGT	ATGGGA	ATGGGT
MH	ATGCAT	ATGCAT	ATGCAT
MI	ATGATT	ATGATT	ATGATT
MK	ATGAAA	ATGAAA	ATGAAA
ML	ATGCTG	ATGTTA	ATGTTG
MN	ATGAAT	ATGAAT	ATGAAT
MP	ATGCCA	ATGCCA	ATGCCT
MQ	ATGCAA	ATGCAA	ATGCAA
MR	ATGCGT	ATGAGA	ATGAGA
MS	ATGAGT	ATGAGT	ATGTCT
MT	ATGACA	ATGACA	ATGACT
MV	ATGGTT	ATGGTT	ATGGTT
MW	ATGTGG	ATGTGG	ATGTGG
MY	ATGTAT	ATGTAT	ATGTAT

Amino acid pair in the C-terminal	E.coli	B.subtilis	S.cerevisiae
AA	GCGGCA	GCAGCG	GCTGCT
AC	GCTTGT	GCTTGT	GCTTGT
AD	GCGGAC	GCTGAT	GCAGAC
AE	GCTGAA	GCTGAA	GCTGAA
AF	GCGTTT	GCTTTT	GCATTT
AG	GCGGGG	GCAGGG	GCAGGT
AH	GCACAT	GCACAT	GCTCAT
AI	GCTATC	GCGATA	GCTATT
AK	GCGAAG	GCAAAA	GCTAAA
AL	GCGCTG	GCGCTG	GCTTTA
AM	GCGATG	GCGATG	GCTATG
AN	GCAAAT	GCAAAC	GCAAAC
AP	GCACCA	GCACCA	GCCCCT
AQ	GCGCAA	GCGCAA	GCTCAG
AR	GCGCGT	GCCCGC	GCAAGG
AS	GCCAGT	GCTTCT	GCTTCA
AT	GCAACG	GCCACA	GCCACA
AV	GCTGTT	GCTGTA	GCTGTT
AW	GCCTGG	GCCTGG	GCTTGG
AY	GCATAT	GCCTAT	GCTTAC
CA	TGCGCA	TGTGCA	TGTGCA
CC	TGTTGT	TGTTGC	TGCTGT
CD	TGTGAC	TGTGAT	TGCGAT
CE	TGCGAA	TGTGAG	TGCGAA
CF	TGTTTT	TGTTTC	TGTTTC
CG	TGCGGC	TGCGGA	TGTGGT
CH	TGTCAT	TGTCAT	TGCCAT
CI	TGTATT	TGTATT	TGTATA
CK	TGTAAA	TGCAAA	TGTAAA
CL	TGCCTG	TGTTTA	TGCCTC
CM	TGTATG	TGTATG	TGTATG
CN	TGTAAC	TGTAAT	TGCAAT
CP	TGTCCG	TGTCCA	TGTCCG
CQ	TGCCAA	TGCCAG	TGCCAG
CR	TGCAGA	TGCAGA	TGCAGA
CS	TGTTCA	TGCAGC	TGTTCA
CT	TGCACG	TGTACA	TGTACG
CV	TGTGTG	TGTGTC	TGTGTT
CW	TGCTGG	TGTTGG	TGTTGG
CY	TGCTAT	TGTTAT	TGCTAC

DA	GACGCC	GATGCT	GACGCT
DC	GATTGC	GACTGC	GACTGT
DD	GATGAT	GATGAC	GATGAT
DE	GATGAA	GATGAA	GATGAG
DF	GATTTT	GATTTT	GATTTT
DG	GACGGT	GACGGC	GATGGA
DH	GATCAT	GATCAT	GATCAT
DI	GATATT	GATATA	GATATT
DK	GATAAG	GATAAA	GACAAA
DL	GATTTA	GATTTG	GATTTA
DM	GATATG	GACATG	GATATG
DN	GATAAC	GACAAC	GATAAT
DP	GATCCA	GACCCG	GACCCT
DQ	GATCAG	GATCAA	GATCAA
DR	GATCGC	GATCGT	GATAGA
DS	GATAGT	GATTCT	GATTCA
DT	GATACA	GATACA	GATACC
DV	GATGTT	GATGTT	GATGTA
DW	GACTGG	GATTGG	GATTGG
DY	GATTAT	GATTAT	GACTAT
EA	GAAGCG	GAAGCG	GAAGCA
EC	GAGTGC	GAGTGT	GAATGC
ED	GAGGAC	GAAGAT	GAAGAT
EE	GAAGAG	GAAGAA	GAAGAA
EF	GAGTTT	GAATTT	GAATTT
EG	GAAGGG	GAAGGA	GAGGGA
EH	GAACAC	GAACAT	GAGCAT
EI	GAGATT	GAAATC	GAAATT
EK	GAAAAA	GAAAAA	GAAAAA
EL	GAACTG	GAATTG	GAATTA
EM	GAAATG	GAAATG	GAAATG
EN	GAAAAT	GAAAAT	GAAAAT
EP	GAACCA	GAGCCT	GAACCA
EQ	GAGCAG	GAACAA	GAACAA
ER	GAGCGA	GAAAGA	GAACGT
ES	GAAAGT	GAATCA	GAATCA
ET	GAAACG	GAAACG	GAAACT
EV	GAGGTG	GAAGTA	GAAGTT
EW	GAGTGG	GAATGG	GAATGG
EY	GAGTAT	GAATAT	GAATAC
FA	TTTGCC	TTTGCT	TTTGCT

FC	TTTTGC	TTCTGC	TTCTGT
FD	TTTGAT	TTTGAC	TTTGAT
FE	TTTGAA	TTTGAA	TTTGAA
FF	TTCTTC	TTTTTC	TTTTTT
FG	TTTGGT	TTTGGC	TTTGGT
FH	TTCCAT	TTTCAT	TTTCAT
FI	TTTATC	TTTATT	TTCATT
FK	TTTAAA	TTTAAA	TTCAAA
FL	TTCCTG	TTTTTA	TTCTTA
FM	TTTATG	TTTATG	TTTATG
FN	TTTAAC	TTTAAT	TTTAAC
FP	TTTCCA	TTTCCG	TTCCCA
FQ	TTCCAA	TTTCAA	TTCCAA
FR	TTCCGC	TTCAGA	TTCAGA
FS	TTTTCG	TTTTCA	TTTAGT
FT	TTCACA	TTCACT	TTTACT
FV	TTTGTT	TTTGTT	TTTGTA
FW	TTTTGG	TTCTGG	TTCTGG
FY	TTTTAT	TTTTAT	TTTTAT
GA	GGTGCC	GGGGCA	GGTGCT
GC	GGCTGT	GGATGT	GGATGT
GD	GGAGAT	GGCGAT	GGTGAC
GE	GGAGAA	GGAGAA	GGAGAG
GF	GGTTTT	GGCTTT	GGATTT
GG	GGGGGT	GGAGGA	GGTGGT
GH	GGTCAT	GGCCAT	GGTCAT
GI	GGGATT	GGGATA	GGCATT
GK	GGGAAA	GGAAAA	GGTAAA
GL	GGCCTG	GGATTG	GGTTTA
GM	GGAATG	GGAATG	GGTATG
GN	GGCAAC	GGAAAT	GGTAAC
GP	GGGCCG	GGGCCG	GGCCCT
GQ	GGGCAG	GGACAG	GGACAA
GR	GGTCGC	GGCCGA	GGTCGT
GS	GGGAGT	GGATCA	GGGAGT
GT	GGAACT	GGTACG	GGGACC
GV	GGCGTT	GGTGTG	GGTGTC
GW	GGCTGG	GGCTGG	GGATGG
GY	GGGTAT	GGTTAT	GGATAT
HA	CACGCC	CATGCA	CATGCA
HC	CATTGC	CACTGC	CATTGT

HD	CACGAT	CATGAT	CATGAC
HE	CATGAG	CATGAA	CATGAA
HF	CATTTC	CATTTT	CATTTT
HG	CACGGT	CATGGA	CATGGT
HH	CACCAT	CATCAT	CACCAT
HI	CATATT	CATATT	CACATT
HK	CACAAA	CATAAA	CATAAA
HL	CATCTT	CATTTA	CATTTG
HM	CATATG	CATATG	CATATG
HN	CACAAT	CATAAC	CATAAC
HP	CATCCA	CATCCA	CACCCT
HQ	CATCAG	CATCAA	CATCAG
HR	CACCGT	CACAGA	CATAGA
HS	CACTCA	CATTCA	CACAGC
HT	CACACC	CATACA	CATACA
HV	CACGTT	CATGTT	CATGTA
HW	CACTGG	CATTGG	CATTGG
HY	CATTAT	CATTAT	CACTAT
IA	ATTGCC	ATCGCA	ATTGCT
IC	ATATGT	ATTTGT	ATTTGT
ID	ATCGAC	ATTGAT	ATTGAT
IE	ATCGAA	ATTGAA	ATTGAA
IF	ATTTTC	ATTTTT	ATTTTT
IG	ATAGGT	ATAGGA	ATTGGA
IH	ATCCAT	ATTCAT	ATTCAT
II	ATTATT	ATTATA	ATTATA
IK	ATTAAA	ATCAAA	ATAAAA
IL	ATTTTG	ATTTTA	ATTTTA
IM	ATAATG	ATTATG	ATTATG
IN	ATTAAT	ATCAAT	ATCAAT
IP	ATTCCA	ATTCCA	ATACCA
IQ	ATTCAG	ATTCAA	ATTCAA
IR	ATTCGC	ATCCGA	ATCAGA
IS	ATCAGC	ATTTCA	ATTAGT
IT	ATCACA	ATTACT	ATAACT
IV	ATTGTT	ATTGTA	ATAGTT
IW	ATATGG	ATATGG	ATATGG
IY	ATATAT	ATTTAT	ATATAC
KA	AAAGCC	AAAGCT	AAGGCT
KC	AAATGC	AAATGT	AAATGT
KD	AAAGAT	AAAGAC	AAGGAC

KE	AAAGAG	AAAGAA	AAAGAA
KF	AAGTTC	AAATTT	AAATTT
KG	AAAGGG	AAAGGA	AAAGGA
KH	AAACAC	AAACAT	AAGCAT
KI	AAAATT	AAAATA	AAAATA
KK	AAAAAA	AAAAAA	AAGAAA
KL	AAGCTG	AAGCTG	AAATTG
KM	AAAATG	AAAATG	AAAATG
KN	AAAAAT	AAAAAC	AAAAAC
KP	AAACCA	AAACCA	AAACCA
KQ	AAACAA	AAACAA	AAACAA
KR	AAGCGT	AAACGA	AAAAGG
KS	AAATCC	AAATCA	AAAAGT
KT	AAAACG	AAAACA	AAAACT
KV	AAAGTA	AAAGTG	AAAGTT
KW	AAATGG	AAATGG	AAATGG
KY	AAATAT	AAATAT	AAATAT
LA	CTGGCG	CTTGCA	TTAGCT
LC	CTGTGC	TTATGC	TTATGT
LD	CTGGAT	TTAGAT	CTAGAT
LE	CTGGAG	CTTGAA	TTGGAA
LF	CTGTTT	TTGTTT	TTGTTT
LG	CTGGGT	TTGGGC	TTGGGA
LH	CTGCAT	TTGCAT	CTACAT
LI	TTGATT	TTAATT	TTGATT
LK	CTGAAA	TTAAAA	TTGAAA
LL	TTGCTG	CTGCTG	TTGTTA
LM	TTGATG	TTGATG	TTAATG
LN	CTGAAC	TTAAAT	TTGAAT
LP	CTGCCG	CTTCCT	CTTCCT
LQ	TTGCAA	CTGCAA	TTACAA
LR	CTGCGT	TTACGT	TTACGT
LS	CTGTCG	CTTTCA	TTATCA
LT	CTGACG	CTCACA	TTGACT
LV	CTGGTG	CTTGTA	CTTGTA
LW	TTGTGG	TTGTGG	CTTTGG
LY	CTGTAC	TTATAT	TTGTAC
MA	ATGGCG	ATGGCA	ATGGCA
MC	ATGTGC	ATGTGC	ATGTGT
MD	ATGGAC	ATGGAC	ATGGAC
ME	ATGGAA	ATGGAA	ATGGAA

MF	ATGTTC	ATGTTT	ATGTTT
MG	ATGGGA	ATGGGG	ATGGGT
MH	ATGCAC	ATGCAC	ATGCAT
MI	ATGATT	ATGATC	ATGATT
MK	ATGAAA	ATGAAA	ATGAAA
ML	ATGCTG	ATGCTT	ATGTTG
MN	ATGAAC	ATGAAT	ATGAAC
MP	ATGCCA	ATGCCG	ATGCCA
MQ	ATGCAG	ATGCAA	ATGCAG
MR	ATGCGT	ATGCGA	ATGAGA
MS	ATGAGT	ATGTCA	ATGTCT
MT	ATGACA	ATGACA	ATGACT
MV	ATGGTG	ATGGTG	ATGGTT
MW	ATGTGG	ATGTGG	ATGTGG
MY	ATGTAT	ATGTAT	ATGTAC
NA	AACGCA	AACGCG	AATGCT
NC	AATTGT	AATTGT	AATTGT
ND	AATGAT	AATGAT	AATGAT
NE	AATGAA	AATGAA	AATGAA
NF	AATTTC	AATTTT	AATTTT
NG	AATGGC	AACGGA	AACGGG
NH	AACCAC	AATCAT	AACCAT
NI	AACATT	AATATA	AATATA
NK	AATAAA	AATAAA	AATAAA
NL	AACCTG	AACTTG	AATTTA
NM	AATATG	AATATG	AATATG
NN	AATAAT	AACAAT	AATAAT
NP	AATCCG	AATCCT	AATCCT
NQ	AATCAA	AATCAA	AATCAA
NR	AACAGA	AACCGC	AATAGA
NS	AACAGC	AATTCA	AATTCT
NT	AATACA	AACACA	AATACA
NV	AACGTT	AATGTA	AATGTT
NW	AACTGG	AACTGG	AATTGG
NY	AACTAC	AATTAT	AATTAT
PA	CCGGCA	CCTGCA	CCAGCT
PC	CCATGC	CCGTGT	CCTTGT
PD	CCTGAC	CCTGAT	CCAGAT
PE	CCTGAA	CCGGAA	CCGGAA
PF	CCGTTC	CCGTTT	CCCTTT
PG	CCGGGA	CCGGGC	CCGGGC

PH	CCGCAT	CCTCAC	CCTCAT
PI	CCTATC	CCTATT	CCCATT
PK	CCCAAA	CCAAAA	CCAAAA
PL	CCGCTG	CCGCTT	CCTTTA
PM	CCAATG	CCGATG	CCAATG
PN	CCGAAT	CCTAAC	CCAAAC
PP	CCGCCA	CCTCCC	CCGCCA
PQ	CCGCAG	CCCCAA	CCACAA
PR	CCGCGT	CCAAGA	CCAAGG
PS	CCTTCC	CCCAGC	CCAAGT
PT	CCAACC	CCGACC	CCTACT
PV	CCTGTT	CCTGTA	CCAGTA
PW	CCCTGG	CCTTGG	CCGTGG
PY	CCCTAT	CCTTAT	CCATAT
QA	CAGGCA	CAAGCA	CAAGCT
QC	CAATGC	CAGTGC	CAGTGT
QD	CAGGAT	CAAGAC	CAAGAT
QE	CAGGAG	CAAGAA	CAAGAA
QF	CAGTTC	CAATTT	CAATTT
QG	CAGGGG	CAAGGA	CAAGGT
QH	CAGCAC	CAGCAT	CAACAT
QI	CAGATT	CAGATA	CAAATA
QK	CAGAAG	CAAAAA	CAAAAA
QL	CAGCTG	CAATTG	CAACTG
QM	CAGATG	CAAATG	CAAATG
QN	CAGAAC	CAAAAC	CAAAAT
QP	CAGCCA	CAGCCA	CAACCT
QQ	CAGCAA	CAGCAA	CAACAA
QR	CAGCGT	CAGAGG	CAAAGA
QS	CAGTCA	CAATCA	CAATCC
QT	CAAACC	CAAACA	CAAACA
QV	CAGGTA	CAAGTA	CAAGTG
QW	CAATGG	CAGTGG	CAATGG
QY	CAGTAC	CAGTAT	CAATAC
RA	CGGGCA	AGAGCA	AGAGCG
RC	CGCTGC	CGGTGC	AGATGT
RD	CGCGAT	AGGGAT	AGAGAT
RE	CGGGAA	AGAGAA	AGAGAG
RF	CGTTTT	AGATTT	AGATTT
RG	CGCGGT	AGAGGA	CGTGGA
RH	CGTCAT	AGACAT	AGACAT

RI	AGAATT	AGAATA	AGAATA
RK	CGTAAA	AGAAAA	AGAAAA
RL	CGCCTG	AGATTA	AGATTA
RM	CGTATG	CGCATG	AGAATG
RN	CGTAAC	CGGAAT	AGAAAC
RP	CGCCCA	CGTCCA	AGACCA
RQ	CGTCAG	AGACAA	AGACAA
RR	CGTCGT	AGAAGA	AGAAGA
RS	CGCAGT	AGAAGC	AGATCA
RT	AGAACC	AGAACG	CGTACT
RV	CGCGTA	CGTGTA	CGTGTA
RW	CGCTGG	CGCTGG	AGATGG
RY	CGTTAC	AGGTAT	AGATAC
SA	AGCGCC	TCTGCA	AGTGCT
SC	TCATGC	TCATGC	TCTTGT
SD	AGCGAT	AGTGAT	AGTGAT
SE	AGCGAA	TCTGAA	TCTGAA
SF	TCGTTC	TCATTT	TCATTT
SG	TCAGGT	AGCGGG	AGCGGC
SH	AGCCAC	TCTCAT	TCTCAT
SI	TCCATC	AGCATA	AGTATA
SK	AGCAAA	TCTAAA	TCTAAA
SL	AGTTTG	TCATTA	TCCTTA
SM	AGCATG	AGCATG	TCAATG
SN	AGCAAC	TCTAAC	TCTAAC
SP	TCACCA	AGCCCG	TCTCCT
SQ	TCGCAG	TCACAA	AGCCAG
SR	AGCCGT	AGCCGA	AGCCGT
SS	TCATCA	TCTTCA	TCATCA
ST	TCCACC	TCAACG	TCCACA
SV	AGCGTT	TCCGTT	TCTGTT
SW	AGCTGG	AGCTGG	AGTTGG
SY	AGTTAT	TCTTAT	TCTTAT
TA	ACAGCC	ACTGCG	ACTGCA
TC	ACATGT	ACATGC	ACATGT
TD	ACAGAT	ACAGAC	ACTGAT
TE	ACTGAA	ACAGAA	ACCGAG
TF	ACGTTC	ACATTC	ACTTTT
TG	ACCGGC	ACAGGA	ACAGGA
TH	ACGCAT	ACACAT	ACTCAC
TI	ACAATC	ACGATT	ACAATA

TK	ACCAAA	ACAAAA	ACCAAA
TL	ACGCTT	ACATTA	ACTTTA
TM	ACAATG	ACAATG	ACTATG
TN	ACTAAC	ACAAAT	ACCAAC
TP	ACACCA	ACGCCT	ACACCC
TQ	ACGCAA	ACACAG	ACTCAA
TR	ACCCGT	ACAAGA	ACGCGT
TS	ACATCA	ACTTCA	ACTTCA
TT	ACAACA	ACAACA	ACTACA
TV	ACGGTG	ACTGTA	ACTGTG
TW	ACCTGG	ACTTGG	ACATGG
TY	ACTTAC	ACCTAT	ACTTAC
VA	GTCGCG	GTTGCT	GTTGCA
VC	GTATGC	GTTTGC	GTCTGT
VD	GTGGAT	GTTGAT	GTTGAT
VE	GTTGAG	GTTGAA	GTTGAG
VF	GTGTTC	GTCTTT	GTATTT
VG	GTTGGG	GTGGGA	GTAGGT
VH	GTTCAT	GTTCAT	GTTCAT
VI	GTGATT	GTTATA	GTTATA
VK	GTGAAA	GTGAAA	GTAAAA
VL	GTGCTG	GTTTTA	GTATTA
VM	GTGATG	GTGATG	GTTATG
VN	GTTAAC	GTCAAT	GTCAAT
VP	GTACCG	GTGCCC	GTACCG
VQ	GTGCAA	GTGCAA	GTACAA
VR	GTGCGT	GTAAGA	GTTAGA
VS	GTCAGT	GTTTCA	GTATCA
VT	GTGACC	GTGACT	GTAACA
VV	GTGGTG	GTTGTT	GTTGTT
VW	GTGTGG	GTGTGG	GTCTGG
VY	GTTTAT	GTATAT	GTTTAT
WA	TGGGCA	TGGGCA	TGGGCA
WC	TGGTGC	TGGTGT	TGGTGT
WD	TGGGAT	TGGGAT	TGGGAT
WE	TGGGAA	TGGGAA	TGGGAA
WF	TGGTTT	TGGTTT	TGGTTT
WG	TGGGGC	TGGGGA	TGGGGT
WH	TGGCAT	TGGCAT	TGGCAT
WI	TGGATT	TGGATA	TGGATA
WK	TGGAAA	TGGAAA	TGGAAA

WL	TGGCTG	TGGCTG	TGGTTA
WM	TGGATG	TGGATG	TGGATG
WN	TGGAAC	TGGAAT	TGGAAT
WP	TGGCCA	TGGCCG	TGGCCA
WQ	TGGCAG	TGGCAG	TGGCAG
WR	TGGCGC	TGGAGA	TGGCGT
WS	TGGAGT	TGGAGC	TGGAGT
WT	TGGACG	TGGACG	TGGACC
WV	TGGGTG	TGGGTT	TGGGTT
WY	TGGTAT	TGGTAT	TGGTAT
YA	TACGCA	TATGCA	TATGCC
YC	TATTGT	TATTGT	TACTGT
YD	TATGAC	TATGAT	TATGAC
YE	TATGAA	TATGAA	TATGAA
YF	TATTTT	TATTTT	TATTTT
YG	TACGGT	TATGGC	TATGGT
YH	TACCAC	TACCAT	TATCAC
YI	TACATT	TATATT	TATATA
YK	TATAAA	TATAAA	TATAAA
YL	TATCTG	TATTTA	TATTTG
YM	TATATG	TATATG	TATATG
YN	TATAAC	TACAAT	TACAAT
YP	TACCCT	TATCCA	TACCCT
YQ	TATCAA	TATCAA	TATCAA
YR	TATCGC	TATAGA	TACAGG
YS	TACAGC	TATTCC	TACTCA
YT	TACACA	TACACA	TACACA
YV	TATGTC	TACGTA	TACGTT
YW	TACTGG	TATTGG	TATTGG
YY	TATTAT	TATTAT	TATTAT
